# Molecularly Distinct Routes of Mitochondrial Ca^2+^ Uptake Are Activated Depending on the Activity of the Sarco/Endoplasmic Reticulum Ca^2+^ ATPase (SERCA)*[Fn FN2]

**DOI:** 10.1074/jbc.M113.462259

**Published:** 2013-04-16

**Authors:** Markus Waldeck-Weiermair, András T. Deak, Lukas N. Groschner, Muhammad Rizwan Alam, Claire Jean-Quartier, Roland Malli, Wolfgang F. Graier

**Affiliations:** From the Institute of Molecular Biology and Biochemistry, Center of Molecular Medicine, Medical University of Graz, 8010-Graz, Austria

**Keywords:** Calcium ATPase, Calcium Intracellular Release, Calcium Signaling, Calcium Transport, Mitochondria

## Abstract

The transfer of Ca^2+^ across the inner mitochondrial membrane is an important physiological process linked to the regulation of metabolism, signal transduction, and cell death. While the definite molecular composition of mitochondrial Ca^2+^ uptake sites remains unknown, several proteins of the inner mitochondrial membrane, that are likely to accomplish mitochondrial Ca^2+^ fluxes, have been described: the novel uncoupling proteins 2 and 3, the leucine zipper-EF-hand containing transmembrane protein 1 and the mitochondrial calcium uniporter. It is unclear whether these proteins contribute to one unique mitochondrial Ca^2+^ uptake pathway or establish distinct routes for mitochondrial Ca^2+^ sequestration. In this study, we show that a modulation of Ca^2+^ release from the endoplasmic reticulum by inhibition of the sarco/endoplasmatic reticulum ATPase modifies cytosolic Ca^2+^ signals and consequently switches mitochondrial Ca^2+^ uptake from an uncoupling protein 3- and mitochondrial calcium uniporter-dependent, but leucine zipper-EF-hand containing transmembrane protein 1-independent to a leucine zipper-EF-hand containing transmembrane protein 1- and mitochondrial calcium uniporter-mediated, but uncoupling protein 3-independent pathway. Thus, the activity of sarco/endoplasmatic reticulum ATPase is significant for the mode of mitochondrial Ca^2+^ sequestration and determines which mitochondrial proteins might actually accomplish the transfer of Ca^2+^ across the inner mitochondrial membrane. Moreover, our findings herein support the existence of distinct mitochondrial Ca^2+^ uptake routes that might be essential to ensure an efficient ion transfer into mitochondria despite heterogeneous cytosolic Ca^2+^ rises.

## Teaser

**Background:** Mitochondria may utilize different proteins to decode high and low cytosolic Ca^2+^.

**Results:** Inhibition of SERCA shifts mitochondrial Ca^2+^ uptake from being UCP3-dependent to Letm1-dependent.

**Conclusion:** Depending on the mode of intracellular Ca^2+^ release, two different mitochondrial Ca^2+^ uptake pathways are engaged.

**Significance:** The dissection of two molecularly distinct mitochondrial Ca^2+^ uptake routes depending on SERCA activity points to the complexity of the mitochondrial Ca^2+^ uptake machinery.

## Introduction

The ability of mitochondria to respond to cytosolic Ca^2+^ elevations is fundamental for cell signaling (1). An accumulation of Ca^2+^ within mitochondria impacts the rate of oxidative phosphorylation (2) and shapes cytosolic Ca^2+^ signals (3, 4). Notably, since excessive Ca^2+^ uptake into mitochondria alters the morphology of the organelle and triggers cell death pathways (5), a proper control of mitochondrial Ca^2+^ sequestration is essential to maintain mitochondrial and cellular homeostasis (6).

Whereas the phenomenon of mitochondrial Ca^2+^ uptake is well characterized as to be accomplished by the so-called mitochondrial Ca^2+^ uniporter, the identification of the actual protein(s) that establish mitochondrial Ca^2+^ uptake is/are still not entirely completed. Moreover, though early patch-clamp studies revealed one Ca^2+^ current in mitoplasts (7), recent reports point to the existence of several distinct mitochondrial Ca^2+^ currents across the inner mitochondrial membrane (IMM)^5^ (8–10), thus, challenging the concept of one sole, ubiquitous, mitochondrial Ca^2+^ uniporter. In agreement with these findings several proteins of the IMM have been identified as putative mitochondrial Ca^2+^ carriers and/or components of mitochondrial Ca^2+^ uptake sites (6, 11). Among these molecules, a 40 kDa protein initially referred to as CCDC109A and then renamed to “mitochondrial calcium uniporter” (MCU) (12, 13) appears to be a promising candidate for a mitochondrial Ca^2+^ channel (6). MCU, which was suggested to represent the Ca^2+^ conducting pore in the IMM, was shown to accomplish mitochondrial Ca^2+^ uptake independently from the source of Ca^2+^. In contrast, the leucine zipper-EF-hand containing transmembrane protein 1 **(**Letm1), which was initially described as a K^+^/H^+^ exchanger in the IMM (14), was also shown to function as a mitochondrial Ca^2+^/H^+^ antiporter (15) that mainly achieved mitochondrial Ca^2+^ accumulation as a result of slow cytosolic Ca^2+^ rises, such as those induced by Ca^2+^ entry via the store-operated Ca^2+^ entry (SOCE) pathway in endothelial and HeLa cells (16). In the same cell types, however, the novel uncoupling proteins 2 and 3 (UCP2/3) were found to contribute primarily to the instant transfer of intracellularly released Ca^2+^ into mitochondria while UCP2/3 did not appear to be engaged in mitochondrial Ca^2+^ uptake upon activation of SOCE (17–19). Such distinct contribution of Letm1 and UCP2/3 to mitochondrial Ca^2+^ uptake might explain the versatility of mitochondria to decode the various patterns of the cytosolic Ca^2+^ signal (20), while the actual molecular function of these proteins remain elusive (6, 21). However, a recent study reports that inhibition of the sarco/endoplasmatic reticulum ATPase (SERCA) abrogates the contribution of UCP3 to mitochondrial Ca^2+^ uptake in HeLa cells (22). Although these findings confirm our previous report, in which mitochondrial Ca^2+^ uptake following SERCA inhibition was shown to be independent of UCP3 (23), the individual interpretation of these data are very different. On one hand, based on their measurements of cytosolic ATP, De Marchi *et al.* concluded that UCP3 is not engaged in mitochondrial Ca^2+^ uptake, but affects the transfer of Ca^2+^ into mitochondria by impacting the SERCA activity via the modulation of mitochondrial ATP generation (22). On the other hand, our group provided experimental evidence, that the contribution of UCP2/3 is independent of the organelles ATP production (17). Hence, upon SERCA inhibition mitochondrial Ca^2+^ uptake is accomplished by a CGP37157-sensitive Ca^2+^ exchanger (23). However, as the potential contribution of a mitochondrial Ca^2+^ exchanger to mitochondrial Ca^2+^ uptake under SERCA inhibition was not evaluated by De Marchi *et al.*, the controversial conclusions remain and await clarification.

Therefore, the present study was designed to solve this controversy. Thus, we employed the same cell model (HeLa cells) and evaluated the contribution of UCP3, Letm1 and MCU to mitochondrial and cytosolic Ca^2+^signaling using a recently developed, mitochondrially targeted red-shifted Ca^2+^ sensor (24) and fura-2/am. This technique allowed us to follow simultaneously respective Ca^2+^ signals in both compartments.

## EXPERIMENTAL PROCEDURES

### 

#### 

##### Chemicals and Buffer Solutions

Cell culture materials were obtained from PAA laboratories (Pasching, Austria). Thapsigargin was purchased from Abcam® (London, UK), Histamine and EGTA were from Sigma (Vienna, Austria). Prior to experiments cells were washed and maintained for 20 min in a HEPES buffered solution composed of (in mm): 138 NaCl, 5 KCl, 2 CaCl_2_, 1 MgCl_2_, 1 HEPES, 2.6 NaHCO_3_, 0.44 KH_2_PO_4_, 0.34 Na_2_HPO_4_, 10 d-glucose, 0.1% vitamins, 0.2% essential amino acids, and 1% penicillin/streptomycin; pH adjusted to 7.4 with NaOH. During the experiments cells were continuously perfused with a Ca^2+^ containing buffer, which consisted of (in mm): 145 NaCl, 5 KCl, 2 CaCl_2_, 1 MgCl_2_, 10 d-glucose, and 10 HEPES; pH adjusted to 7.4 with NaOH. In experiments where a Ca^2+^-free solution was applied to the cells, the CaCl_2_ was replaced with 1 mm EGTA.

##### siRNAs and Approval of Their Respective Knock-down Efficiency

The siRNAs against human MCU, UCP2/3 and Letm1 were obtained from Microsynth (Balgach, Switzerland) and their nucleotide sequences (5′-3′) were as follows: si1-hMCU: GCCAGAGACAGACAAUACUtt; si2-hMCU: GGAAAGGGAGCUUAUUGAAtt; si1-hLetm1: UCCACAUUUGAGACUCAGUtt; si2-hLetm1: AUGUUCCAUUUGGCUGCUGtt; si-hUCP2: GCACCGUCAAUGCCUACAAtt; si-hUCP3: GGAACUUUGCCCAACAUCAtt.

For controls, a scrambled siRNA was used: UUCUCCGAACGUGUCACGUtt. Although all siRNAs used in this study have been previously approved to exhibit reliable gene knock-down efficiency (16), their efficiency was again verified by quantitative RT-PCR in HeLa as previously described (25).

Total RNA was isolated from control and target siRNA treated HeLa cells using a RNA isolation kit (PEQLAB Biotechnologie GmbH, Erlangen, Germany). Reverse transcription was carried out using a cDNA synthesis kit from Applied Biosystems. The efficiency of siRNA was validated by performing Real time PCR using QuantiFast SYBR Green RT-PCR kit (Qiagen, Hilden, Germany) on LightCycler 480 (Roche Diagnostics, Vienna, Austria). RNA polymerase II (RPOL2) was used as housekeeping control. Primers for RPOL2, UCP2, UCP3, MCU, and LETM1 were obtained from Invitrogen (Vienna, Austria) and there sequences (5′-3′) are as follows: RPOL2 for: CATTGACTTGCGTTTCCACC, RPOL2 rev: ACATTTTGTGCAGAGTTGGC, UCP2 for: TCCTGAAAGCCAACCTCATG, UCP2 rev: GGCAGAGTTCATGTATCTCGTC, UCP3 for: AGAAAATACAGCGGGACTATGG, UCP3 rev: CTTGAGGATGTCGTAGGTCAC, MCU for: TTCCTGGCAGAATTTGGGAG, MCU rev: AGAGATAGGCTTGAGTGTGAAC, Letm1 for: TGTTCTTCAAGGCCATCTCC, Letm1 rev: TGTTGCTGTGAAGCTCTTCC. The expression data were analyzed by ΔΔCt method as described previously (25). Knock-down efficiency was in the same range than previously reported for endothelial cells (16).

##### Cell Culture and Transfection

HeLa cells were cultured as described previously (24). Briefly, cells were grown in Dulbeccos's Modified Eagle Medium (Sigma, Vienna, Austria) containing 10% fetal bovine serum, 100 units/ml penicillin, and 100 μg/ml streptomycin and were plated on 30-mm glass coverslips. At 60–80% confluency, cells were transfected with 2 μg (per 30-mm well) of plasmid DNA encoding 4mtD1GO-Cam (24) alone or in combination with 100 μm siRNA using 4 μg/well TransFast^TM^ transfection reagent (Promega, Madison, WI) in 0.5 ml of serum and antibiotic-free transfection medium. Cells were maintained in a humidified incubator (37 °C, 5% CO_2_, 95% air) for 16–20 h before changing back to complete RPMI 1640 medium. All experiments were performed either 48 h or 72 h after transfection.

##### Simultaneous Cytosolic and Mitochondrial Ca^2+^ Measurements

4mtD1GO-Cam (24) transfected HeLa cells were loaded with 2 μm fura-2/AM (TEFLabs, Austin, TX) for 45 min prior to the experiments. Co-imaging of fura-2 and the 4mtD1GO-Cam was achieved with a digital wide field imaging system, the Till iMIC (Till Photonics Graefelfing, Germany) using a 40× objective (alpha Plan Fluar 40×, Zeiss, Göttingen, Germany). For illumination of fura-2 and the 4mtD1GO-Cam an ultra fast switching monochromator, the Polychrome V (Till Photonics) equipped with an excitation filter (E500spuv, Chroma Technology Corp., Rockingham, Vermont) and a dichroic filter (495dcxru, Chroma Technology Corp) was used. fura-2 was excited alternatively at 340 nm and 380 nm and the red-shifted mitochondrial-targeted cameleon was excited at 477 nm, respectively. Emitted light was simultaneously collected at 510 nm (fura-2 and GFP of GO-Cam) and at 560 nm (FRET-channel of GO-Cam) using a single beam splitter design (Dichrotome, Till Photonics) that was equipped with a dual band emission filter (59004m ET Fitc/Tritc Dual Emitter, Chroma Technology Corp.) and a second dichroic filter (560dcxr, Chroma Technology Corp.). Images were recorded with a charged-coupled device (CCD) camera (AVT Stringray F145B, Allied Vision Technologies, Stadtroda, Germany). For the data acquisition and the control of the digital fluorescence microscope the live acquisition software (LA) version 2.0.0.12 (Till Photonics) was used. Post-acquisition image analysis was performed on MetaMorph 7.7.0.0 (Visitron Systems, Puchheim, Germany).

##### Statistics

Data shown represent the mean ± S.E., where *n* reflects the number of cells. Statistical analyses were performed with unpaired Student's *t* test, and *p* < 0.05 was considered to be significant.

## RESULTS

### 

#### 

##### SERCA Inhibition Slows Down the IP_3_-mediated Transfer of Ca^2+^ into Mitochondria

**FIGURE 1.**
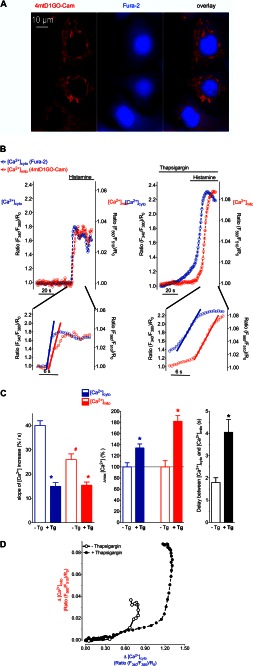
**SERCA inhibition prior to IP_3_-mediated Ca^2+^ release impacts the kinetics of Ca^2+^ signals and coupling between [Ca^2+^]_cyto_ and [Ca^2+^]_mito_.** *A*, HeLa cells expressing the mitochondrial Ca^2+^ sensor 4mtD1GO-Cam (*red*) were loaded with fura-2/AM (*blue*). Images were taken with a fully automated fluorescence microscope using a camera binning of 4. *B*, *upper panels:* representative traces of cytosolic (*blue curves*) and mitochondrial (*red curves*) Ca^2+^ signals in HeLa cells upon stimulation with 100 μm histamine in the absence of Ca^2+^ (*left upper panel*). SERCA inhibition was achieved by using 1 μm thapsigargin that was added 40 s prior to cell treatment with histamine (*right upper panel*). Data are expressed as normalized ratios: (F_340_/F_380_)/R_0_ for [Ca^2+^]_cyto_ and (F_560_/F_510_)/R_0_ for[Ca^2+^]_mito._ R_0_ was calculated from basal ratio values for each individual cell respectively. *Lower panels:* zoom-in of *upper panels* showing which part was used for calculating the slope of Ca^2+^ increase. Following onset, each curve was fitted with linear regression (*bold lines*) to assess maximal slope of cytosolic and mitochondrial [Ca^2+^] elevations. *C*, statistical evaluation of the Ca^2+^ signals presented in *panel B. Left panel:* columns represent the maximal slopes of [Ca^2+^]_cyto_ (*blue columns*) and [Ca^2+^]_mito_ (*red columns*) increases upon histamine stimulation in the absence (*white columns*, *n* = 18) or presence of thapsigargin pretreatment (*filled columns*, *n* = 15). *Middle panel:* columns represent the average of maximum delta ratios in the absence (*white columns*) or prescence of thapsigargin preincubation (*filled columns*). Cytosolic and mitochondrial [Ca^2+^] elevation in response to cell treatment with 100 μm histamine was defined as 100%, respectively (*B*, *left panel*). *Right panel:* lag times in seconds between cytosolic and respective mitochondrial Ca^2+^ rises in the absence of thapsigargin (*white column*, *n* = 18) and upon pretreatment with thapsigargin (*black column*, *n* = 15). *, *p* < 0.05 *versus* in the absence of thapsigargin (−Tg), #, *p* < 0.05 *versus* [Ca^2+^]_cyto_ in the absence of thapsigargin (−Tg). *D*, representative temporal correlations between histamine-induced (100 μm) changes of [Ca^2+^]_cyto_ (*x* axis) and [Ca^2+^]_mito_ (*y* axis) in the absence of thapsigargin (continuous line with *open circles*) and upon pretreatment with the SERCA inhibitor (*dotted line with filled circles*).

[Ca^2+^]_cyto_ and [Ca^2+^]_mito_ were simultaneously measured using Fura-2/AM-loaded cells that transiently expressed 4mtD1GO-Cam, a recently developed red shifted genetically encoded Ca^2+^ probe targeted to the mitochondrial matrix (Fig. 1*A*). This approach allowed an accurate temporal correlation of changes in [Ca^2+^]_cyto_ with [Ca^2+^]_mito_. Stimulation with the IP_3_-generating agonist histamine in the absence of extracellular Ca^2+^ induced a fast increase of both cytosolic and mitochondrial Ca^2+^ levels (Fig. 1*B*, *left panel*), indicating an efficient transfer of Ca^2+^ from the endoplasmic reticulum (ER) into mitochondria. The Ca^2+^ signal in the cytosol occurred slightly faster than the respective Ca^2+^ elevation within mitochondria of same single individual cells (Fig. 1, *B*, *left panel* & *C*). Pretreating the cells with the SERCA inhibitor thapsigargin 40 s prior to the addition of histamine, enhanced the cytosolic Ca^2+^ elevation (Fig. 1, *B*, *right panel* & *C*). Notably, in the presence of thapsigargin, [Ca^2+^]_cyto_ started to increase slowly, indicating Ca^2+^ leakage from the ER. This weak thaspsigargin-induced cytosolic Ca^2+^ signal was not accompanied by a significant elevation in [Ca^2+^]_mito_ (Fig. 1*B*, *right panel*). Subsequent addition of histamine evoked a further pronounced rise of both [Ca^2+^]_cyto_ and [Ca^2+^]_mito_. However, these signals increased with a slower kinetics compared with respective Ca^2+^ elevations in the absence of the SERCA inhibitor (Fig. 1, *B* & *C*). In addition, the time gap between the histamine induced rise of [Ca^2+^]_cyto_ and the respective mitochondrial Ca^2+^ signal was considerably extended in the presence of thapsigargin (Fig. 1, *B* & *C*), indicating that SERCA inhibition decelerates the transfer of Ca^2+^ into mitochondria upon IP_3_-mediated Ca^2+^ release. A correlation between changes of [Ca^2+^]_cyto_ and respective Ca^2+^ signals within mitochondria showed that in the presence of thapsigargin almost twice as much cytosolic Ca^2+^ was elevated, until mitochondrial Ca^2+^ uptake was activated (Fig. 1*D*).

These protocols that illustrate the distinct kinetics of the compartmental Ca^2+^ rises and coupling between [Ca^2+^]_cyto_ and [Ca^2+^]_mito_ were subsequently used to investigate the contribution of the individual proteins that have been proposed to be involved in mitochondrial Ca^2+^ uptake (*i.e.* UCP3, Letm1, and MCU).

##### SERCA Inhibition Switches Mitochondrial Ca^2+^ Uptake from a UCP3-dependent into a Letm1-dependent Mode

We speculated that the SERCA-dependent differences in the kinetics of mitochondrial Ca^2+^ signals probably reflect the involvement of distinct mitochondrial Ca^2+^ uptake routes. Therefore, we performed experiments, in which the contribution of the mitochondrial proteins UCP2/3, Letm1 and MCU to mitochondrial Ca^2+^ uptake in various protocols was investigated by diminution of these proteins with a transient transfection of the respective siRNA. The siRNAs against UCP2/3, Letm1, and MCU have already been validated to specifically and significantly reduce mRNA level of the respective proteins (16).

**FIGURE 2.**
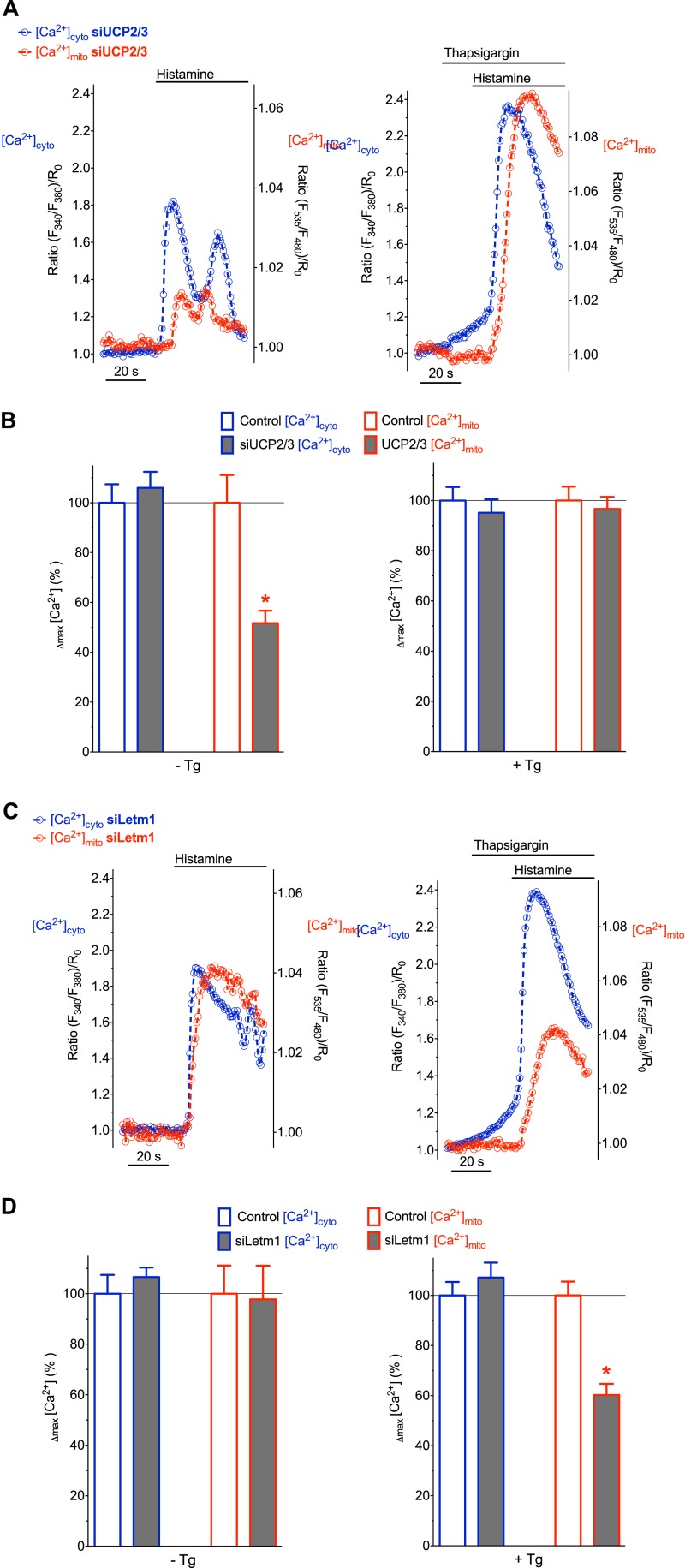
**Depending on the SERCA activity either UCP2/3 or Letm1 contribute to mitochondrial Ca^2+^ uptake.** *A*, representative recordings of [Ca^2+^]_cyto_ (*blue*) and [Ca^2+^]_mito_ (*red*) in single individual HeLa cells transfected with siRNAs against UCP2/3. Cells were treated with 100 μm histamine alone (*left panel*) or together with 1 μm thapsigargin (*right panel*). *B*, column statistics of histamine-evoked cytosolic (*blue-bordered columns*) and mitochondrial (*red-bordered columns*) Ca^2+^ signals in HeLa cells transfected either with scrambled siRNA (control, *white columns*) or with siRNA against UCP2/3 (UCP2/3, *gray columns*). Experiments were performed in the absence (*left panel*, *n* = 18 for control and *n* = 19 for siUCP2/3) or in the presence of 1 μm thapsigargin pretreatment (*right panel*, *n* = 15 for control and *n* = 17 for siUCP2/3). The delta maximum of normalized cytosolic and mitochondrial Ca^2+^ signals were defined as 100% under control conditions (*i.e.* cells transfected with scrambled siRNA as shown in Fig. 1*C*) both in the absence (*left panel*) and presence (*right panel*) of thapsigargin, *, *p* < 0.05 *versus* Control [Ca^2+^]_mito_ *C*, simultaneous, representative recordings of [Ca^2+^]_cyto_ (*blue*) and [Ca^2+^]_mito_ (*red*) in HeLa cells transfected with siRNA against Letm1. Ca^2+^ signals were evoked with 100 μm histamine in the absence (*left panel*) or presence (*right panel*) of 1 μm thapsigargin. *D*, column statistics of cytosolic and mitochondrial Ca^2+^ signals in HeLa cells transfected with siRNA against Letm1 (*gray columns*) without thapsigargin (*left panel*, *n* = 19) and upon pretreatment with thapsigargin (*right panel*, *n* = 17). *White columns* represent control conditions as indicated in *panel B*. *, *p* < 0.05 *versus* Control [Ca^2+^]_mito_.

In line with previous studies (17, 18, 22), a transient knock-down of UCP3 significantly reduced the histamine-induced mitochondrial Ca^2+^ signal, while the respective cytosolic Ca^2+^ elevation was only minimally affected (Fig. 2, *A*, *left panel* & *B*). As recently demonstrated (22), SERCA inhibition with thapsigargin, which was added shortly before histamine, abolished the effect of UCP3 knock-down on [Ca^2+^]_mito_ (Fig. 2, *A*, *right panel* & *B*).

Next we performed analogous experiments with cells, in which Letm1 was silenced (Fig. 2, *C* & *D*). Mitochondrial Ca^2+^ sequestration upon IP_3_-mediated Ca^2+^ mobilization in the absence of thapsigargin was not affected in cells that were treated with siRNA against Letm1 (Fig. 2, *C*, *left panel* & *D*). In contrast, if SERCA activity prior to the addition of the agonist was blocked, diminution of Letm1 strongly reduced the histamine-induced mitochondrial Ca^2+^ signal (Fig. 2, *C*, *right panel* & *D*). Notably, the siRNA-mediated knock-down of UCP2/3, Letm1, and MCU neither affected the mitochondrial membrane potential (supplemental Fig. S1), nor the capacity of mitochondria to extrude Ca^2+^ (supplemental Fig. S2). These data indicate that SERCA inhibition switches the mode of mitochondrial Ca^2+^ uptake from a Letm1-independent to a Letm1-dependent one and, hence, explain the lacking contribution of UCP2/3 to mitochondrial Ca^2+^ uptake under conditions of SERCA inhibition.

##### MCU Contributes to Mitochondrial Ca^2+^ Uptake Independently from SERCA Activity and, Hence, the Mode of Ca^2+^ Mobilization

**FIGURE 3.**
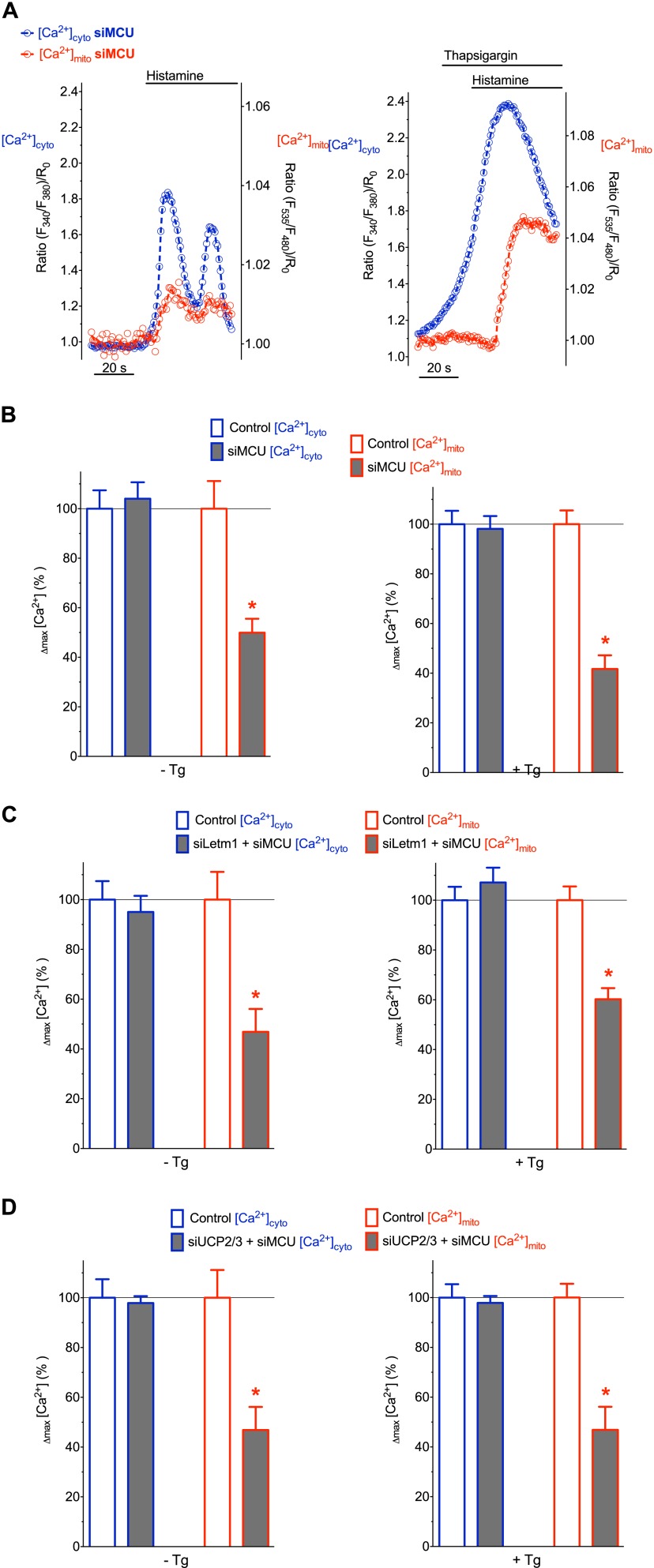
**Transient knock-down of MCU results in a diminished mitochondrial Ca^2+^ uptake independently from SERCA activity.** *A*, representative curves of [Ca^2+^]_cyto_ (*blue*) and [Ca^2+^]_mito_ (*red*) in HeLa cells transfected with siRNA against MCU that were treated with 100 μm histamine in the absence of thapsigargin (*left panel*) and upon pretreatment with the SERCA inhibitor (*right panel*). *B*, column statistics of cytosolic (*blue border columns*) and mitochondrial Ca^2+^ (*red-bordered columns*) signals in control HeLa cells (*white columns*) and cells transfected with siRNA against MCU (*gray columns*) without thapsigargin (*left panel*, *n* = 24) and upon pretreatment with thapsigargin (*right panel*, *n* = 14). *White columns* represent control conditions (defined as 100%) as indicated in Fig. 2. *, *p* < 0.05 *versus* Control [Ca^2+^]_mito_. *C*, HeLa cells transfected with siRNA against Letm1 and MCU were stimulated with histamine in the absence (*left panel*, *n* = 18) and presence (*right panel*, *n* = 14) of thapsigargin in experimental conditions indicated in *panel A. D*, HeLa cells transfected with siRNA against UCP2/3 and MCU were stimulated with histamine in the absence (*left panel*, *n* = 27) and presence (*right panel*, *n* = 14) of thapsigargin in experimental conditions indicated in *panel A*.

To investigate the participation of MCU in the transfer of intracellularly released Ca^2+^ into mitochondria, respective experiments were performed with MCU-depleted cells. The knock-down of MCU negligibly altered cytosolic Ca^2+^ signals in response to cell stimulation (Fig. 3*A* & Fig. 5*B*). However, respective mitochondrial Ca^2+^ signals were strongly diminished in cells depleted of MCU (Fig. 3, *A* & *B*). Notably, the inhibitory effect of MCU knock-down on mitochondrial Ca^2+^ accumulation was independent of the absence or presence of thapsigargin (Fig. 3), indicating that MCU facilitates mitochondrial Ca^2+^ uptake independently of SERCA activity and, hence, the mode of Ca^2+^ mobilization.

A simultaneous knock-down of either MCU and UCP2/3 or MCU and Letm1 did not further reduce mitochondrial Ca^2+^ uptake under the various conditions (Fig. 3, *C* and *D* & supplemental Fig. S3), thus, pointing to a functional interaction between these putative contributors of mitochondrial Ca^2+^ uptake.

##### SERCA Inhibition Following IP_3_-mediated Ca^2+^ Release Increases SOCE and Also Abrogates the Contribution of UCP2/3 to MCU-mediated Mitochondrial Ca^2+^ Uptake, While Letm1 Gets Involved

SERCA inhibition prior to cell stimulation with histamine partially emptied the internal Ca^2+^ store that results in a decelerated, but higher cytosolic Ca^2+^ signal and a greater delay of the Ca^2+^ transfer into the mitochondria (Fig. 1*B*, *right panel*) suggesting a shift in the mode/route of mitochondrial Ca^2+^ uptake.

**FIGURE 4.**
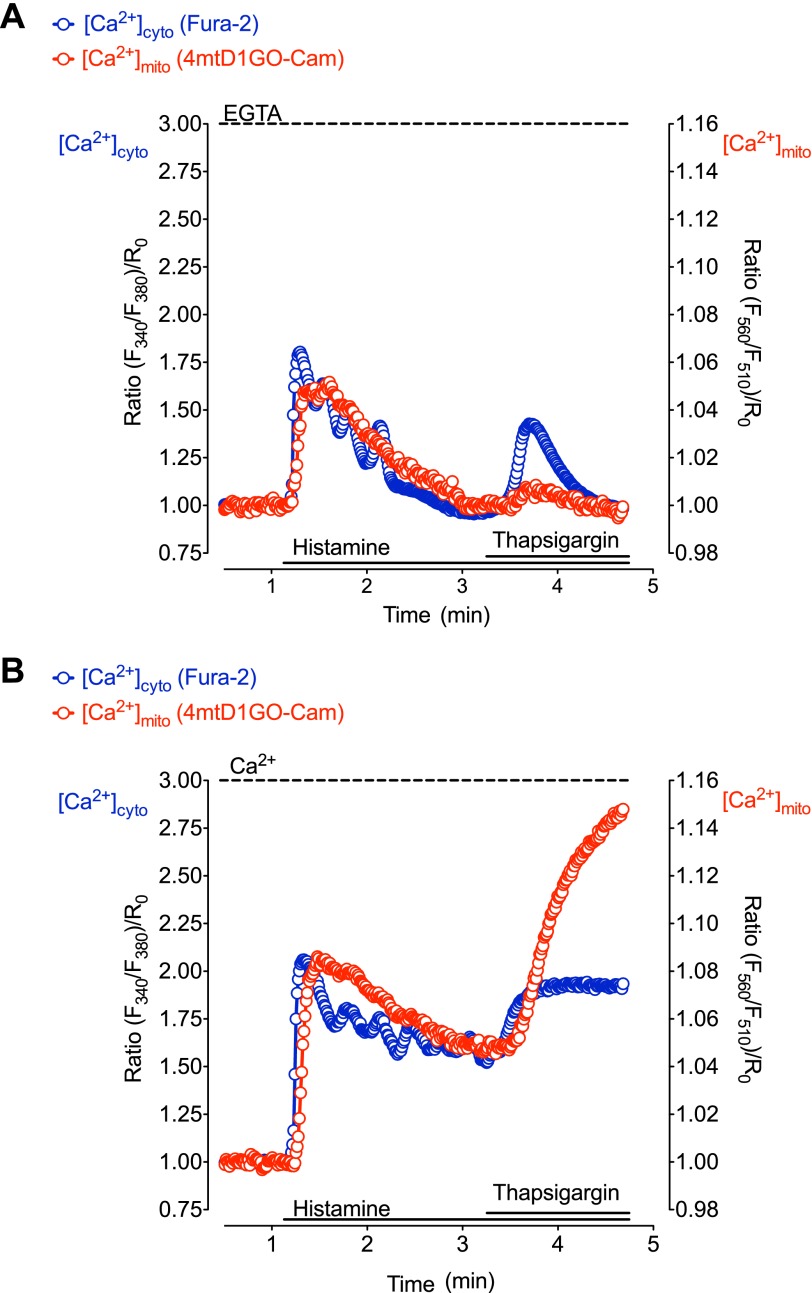
**SERCA inhibition during cell stimulation with an IP_3_-generating agonist enhances [Ca^2+^]_cyto_ and [Ca^2+^]_mito_.** Representative curves demonstrating simultaneous measurement of [Ca^2+^]_cyto_ (*blue*) and [Ca^2+^]_mito_ (*red*) in HeLa cells loaded with fura-2/AM and transiently transfected with 4mtD1GO-Cam. *A*, cells were treated with 100 μm histamine for 2 min before the addition of 1 μm thapsigargin in the absence of extracellular Ca^2+^. *B*, cells were treated with 100 μm histamine for 2 min before the addition of 1 μm thapsigargin in the presence of 2 mm Ca^2+^ in the extracellular medium.

To test whether or not this change in mitochondrial Ca^2+^ uptake mode/route also occurs under condition in which the cell is already stimulated by an IP_3_-generating agonist, we performed different experimental protocols, in which thapsigargin was added after histamine. If cells were continuously exposed to the IP_3_ generating agonist in the absence of extracellular Ca^2+^, the subsequent SERCA inhibition transiently elevated [Ca^2+^]_cyto_ (Fig. 4*A*). This transient thapsigargin-induced increase of cytsolic Ca^2+^ levels evoked only small changes of [Ca^2+^]_mito_. In contrast, SERCA inhibition following IP_3_-mediated Ca^2+^ release in the presence of extracellular Ca^2+^-induced prominent, longer-lasting elevations of both [Ca^2+^]_cyto_ and [Ca^2+^]_mito_ (Fig. 4*B*), thus, highlighting the involvement of SOCE that promotes mitochondrial Ca^2+^ accumulation. The latter protocol was further used to test the contribution of UCP3, Letm1, and MCU to mitochondrial Ca^2+^ sequestration under these conditions of Ca^2+^ mobilization.

**FIGURE 5.**
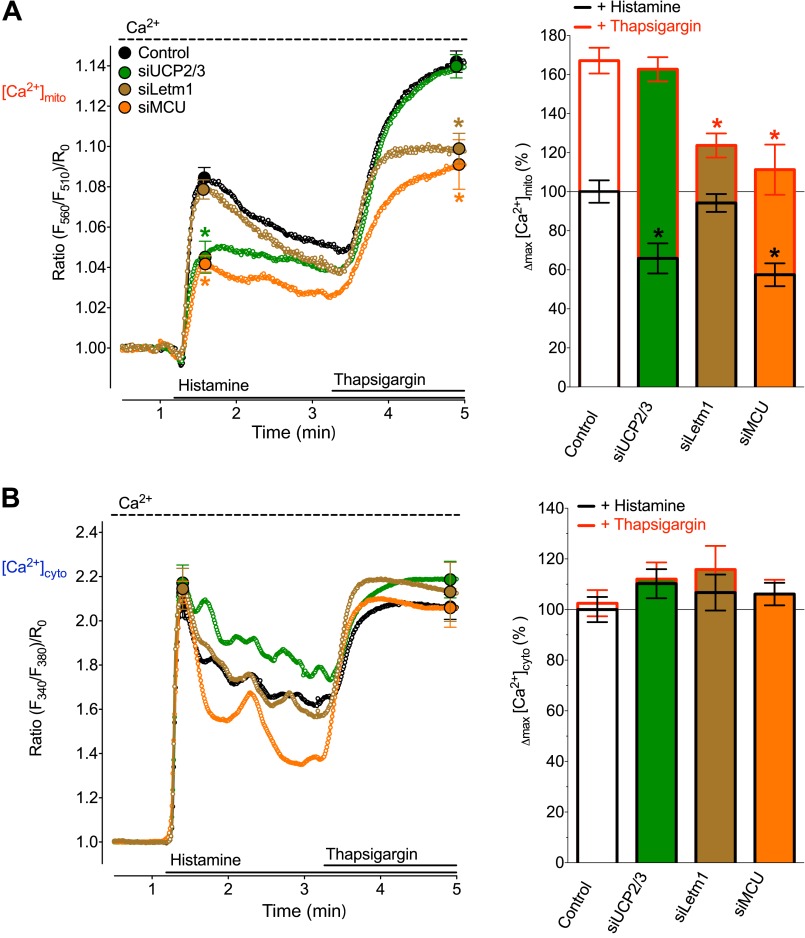
**SERCA inhibition during cell stimulation with histamine switches mitochondrial Ca^2+^ uptake from being UCP2/3 and MCU dependent to an UCP2/3 independent, but Letm1 and MCU-dependent mode.** HeLa cells were either transfected with an inoperative Control siRNA (*black curves* and *white columns*, *n* = 18), or siRNAs against UCP2 and UCP3 (*green curves* and *green columns*, *n* = 18), or Letm1 (*brown curves* and *brown columns*, *n* = 20) or MCU (*orange curves* and *orange columns*, *n* = 19). *A*, average curves (*left panel*) and statistical data (*right panel*) of [Ca^2+^]_mito_ signals measured with 4mtD1GO-Cam upon cell treatment with 100 μm histamine and the subsequent addition of 1 μm thapsigargin in the presence of 2 mm Ca^2+^. *, *p* < 0.05 *versus* respective Controls (*B*). Respective cytosolic Ca^2+^ curves (*left panel*) and statistical analysis of Δmax [Ca^2+^]_cyto_ values (*right panel*) from Fura-2 signals of HeLa cells that were treated as indicated in Fig. 1*A*.

Consistent with our previous work and the experiments in the absence of extracellular Ca^2+^ (Fig. 2, *B* and *D*) cells depleted of UCP3 showed greatly reduced mitochondrial Ca^2+^ accumulation in response to histamine (Fig. 5*A*). The subsequent addition of thapsigargin evoked a substantial rise of [Ca^2+^]_mito_ that was not affected by the diminution of UCP3.

In contrast, knock-down of Letm1 negligibly influenced mitochondrial Ca^2+^ uptake that was elicited by histamine, whereas [Ca^2+^]_mito_ was significantly reduced in response to a subsequent SERCA inhibition (Fig. 5*A*). These findings demonstrated that in protocols in which SERCA was blocked after the initiation of IP_3_-mediated Ca^2+^ release, mitochondrial Ca^2+^ uptake also switched from an UCP3-reliant to a Letm1-dependent mode.

Consistent with previous experiments, cells that were treated with siRNA against MCU showed attenuated mitochondrial Ca^2+^ signals in response to histamine and to the subsequent addition of thapsigargin (Fig. 5*A*). Notably, the initial cytosolic Ca^2+^ peak and the thapsigargin induced rise remained unaffected under all conditions (Fig. 5*B*).

## DISCUSSION

**FIGURE 6.**
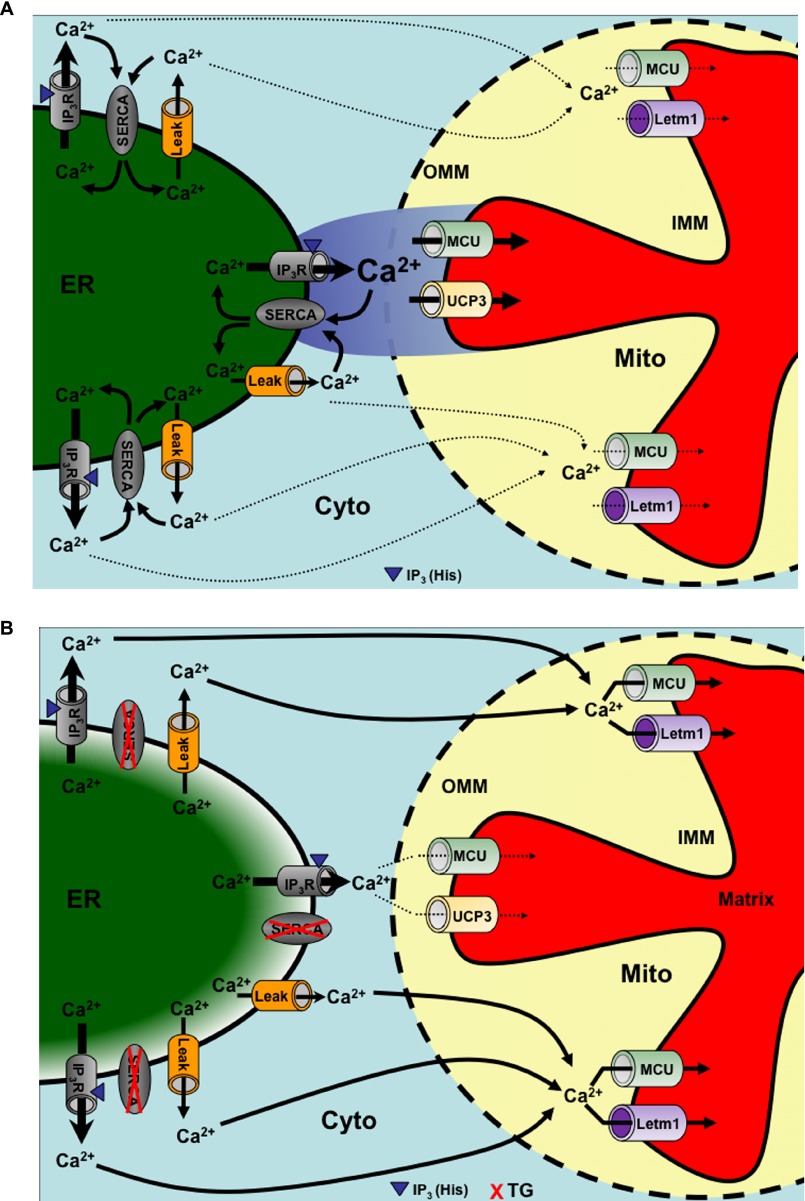
**Schematic illustration of a hypothetical switch of mitochondrial Ca^2+^ uptake by SERCA inhibition upon IP_3_-mediated Ca^2+^ release.** *A*, SERCA activity counteracting ER Ca^2+^ leakage and recycling Ca^2+^ into the ER supports the generation of high Ca^2+^ micro-domains upon IP_3_-mediated ER Ca^2+^ release. Under these conditions Ca^2+^ hot spots at close sites of mitochondrial Ca^2+^ uptake are sensed by UCP2/3 and MCU, which accomplish the transfer of Ca^2+^ across the IMM. *B*, impaired SERCA activity yields partial ER Ca^2+^ depletion counteracting the generation of high Ca^2+^ micro-domains at mitochondrial contact sites. Under these conditions the global slow cytosolic Ca^2+^ elevation is partially transferred into mitochondria by Letm1 and MCU. *Arrows* indicate Ca^2+^ fluxes.

Our present data reveal that IP_3_-mediated, rapid Ca^2+^ rises, that are associated with the generation of high Ca^2+^ micro-domains at the surface of mitochondria (26) were transferred into these organelles via an UCP3- and MCU-dependent but Letm1-independent pathway (Fig. 6*A*). However, if the mode of Ca^2+^ mobilization was decelerated by SERCA inhibition, which increased ER Ca^2+^ leakage and, thus, probably attenuated the inter-organelle Ca^2+^ micro-domains, mitochondria slowly accumulated Ca^2+^ via an UCP3-independent, but Letm1- and MCU-reliant route (Fig. 6*B*).

Basically, our current findings indicate that a modification of the IP_3_-mediated Ca^2+^ release by SERCA inhibition significantly alters the molecular characteristics of mitochondrial Ca^2+^ uptake. At first glance, these findings are not surprising, considering the central role of ER Ca^2+^ pumps in the control of the cellular Ca^2+^ homeostasis (27, 28). Virtually, in all cells, SERCA activity is necessary to maintain high Ca^2+^ levels within the ER by counteracting Ca^2+^ leakage and to restore Ca^2+^ upon events of ER Ca^2+^ release (29, 30). In line with these reports, our measurements show that a combination of the SERCA inhibitor with the IP_3_-generating agonist boosts the total increases in global cytosolic and mitochondrial Ca^2+^ signals, thus, further supporting the hypothesis that during IP_3_-mediated ER depletion, SERCA activity counteracts cytosolic Ca^2+^ rises under control conditions (31).

One possible explanation for this obvious switch in the mode of mitochondrial Ca^2+^ uptake is an attenuation of high Ca^2+^ micro-domains in the inter-organelle gap between the ER and mitochondria upon inhibition of SERCA. High Ca^2+^ micro-domains at ER-mitochondria contact sites were supposed to be fundamental for the activation of the low Ca^2+^ sensitive mitochondrial Ca^2+^ uniport upon IP_3_-mediated ER Ca^2+^ release (32–34). In sophisticated studies the existence of such high Ca^2+^ micro-domains, also referred to as Ca^2+^ hot spots, on sites of mitochondrial Ca^2+^ uptake has been recently demonstrated (26, 35). Although we did not measure local Ca^2+^ hot spots, based on the clear effects of the SERCA activity on global cytosolic Ca^2+^ signals we reported herein, it is likely that an acute deactivation of ER Ca^2+^ pumps prior to or during the activation of the IP_3_-mediated pathway also considerably impedes the formation of such local Ca^2+^ domains. In agreement with this assumption, our data demonstrate that a loss of SERCA activity in HeLa cells instantly increases a leak of Ca^2+^ from the ER resulting in a slow and moderate cytosolic Ca^2+^ elevation that is accompanied with a tiny but measurable increase in [Ca^2+^]_mito_. Due to the slightly lower Ca^2+^ affinity of the cameleon probe used for measuring [Ca^2+^]_mito_ (1.53 μm, (24)) than that of fura-2 (224 nm, (36)), the mitochondrial Ca^2+^ elevation upon thapsigargin might be underestimated. However, these data clearly show that despite the massive and pronounced ER depletion and its associated slow, but considerable cytosolic Ca^2+^ elevation, mitochondrial Ca^2+^ elevation remains small. Accordingly, the ER Ca^2+^ leakage might be locally facilitated by neighboring mitochondria that sequester and buffer the leaked Ca^2+^ from the inter-organelle gap and, thus, maintain the great Ca^2+^ gradient. Consequently, such phenomenon might result in an accelerated local ER Ca^2+^ depletion in ER regions that are in the vicinity of mitochondria. Such scenario would explain the decelerated cytosolic Ca^2+^ rise in response to IP_3_-mediated Ca^2+^ mobilization upon the short preincubation with a SERCA inhibitor. Under such conditions, the IP_3_-triggered formation of inter-organelle Ca^2+^ hot spots is hampered, thus, a mitochondrial Ca^2+^ carrier such as UCP3 that, might require high Ca^2+^ domains to be activated due to its low Ca^2+^ sensitivity (19) is inactive.

In addition, the increased Ca^2+^ leak from the ER upon SERCA inhibition might *per se* impact the transfer of Ca^2+^ into mitochondria. Such a scenario is feasible, as it was shown that an increased ER Ca^2+^ leakage in cells expressing an inactive truncated version of SERCA1 inhibited mitochondrial movements and increased ER-mitochondria contact sites, which consequently led to mitochondrial Ca^2+^ overload (37). However, our data showed that the thapsigargin induced Ca^2+^ leak in HeLa cells is only slowly and moderately transferred into mitochondria. Nevertheless, the increased Ca^2+^ leak upon SERCA inhibition might indeed affect the local organization and architecture of ER-mitochondria contact sites and, thus, the mode of mitochondrial Ca^2+^ uptake (38–41). Notably, using genetically encoded linker proteins that allowed a definite tethering of the ER to mitochondria indicated that the distance of the gap between both organelles is determent for the ability of mitochondria to sense Ca^2+^ hot spots upon ER Ca^2+^ release (42).

Irrespective of high local Ca^2+^ signals at inter-organelle junctions between mitochondria and the ER, mitochondria have been shown to accomplish also the uptake of smooth, moderate cytosolic Ca^2+^ elevations (18, 32–34). The coexistence of both rapid mitochondrial uptake of high Ca^2+^ micro-domains and mitochondrial Ca^2+^ sequestration of global slow rising Ca^2+^ signals is consistent with our data presented in this study. Moreover, our findings highlight a clear delay between cytosolic and respective mitochondrial Ca^2+^ signals, particularly if the IP_3_-mediated cytosolic Ca^2+^ elevation was decelerated by SERCA inhibition. Accordingly, we assumed that the slow and delayed mitochondrial Ca^2+^ accumulation in the presence of the SERCA inhibitor exhibits a specific mode of Ca^2+^ transfer across the IMM, which is distinct from mitochondrial Ca^2+^ uptake during fast IP_3_-mediated ER Ca^2+^ release. This “slow” presumably highly sensitive type of mitochondrial Ca^2+^ accumulation in the presence of thapsigargin might be comparable with mitochondrial uptake of Ca^2+^ entering the cell via the SOCE pathway (19). Notably, it was shown that mitochondria in HeLa cells are not exposed to Ca^2+^ hot spots in response to SOCE (26). These findings are also consistent with our previous study using endothelial cells, which showed a diffusion dependent and, hence, slow mode of mitochondrial Ca^2+^ sequestration if Ca^2+^ was mobilized via the SOCE pathway (18). In this endothelial cell model we unveiled that the slow mode of mitochondrial Ca^2+^ sequestration upon SOCE especially requires Letm1, while UCP2/3 contributed exclusively to fast mitochondrial uptake of Ca^2+^ that was mobilized via the IP_3_ pathway (16). These findings are in line with the observed switch of mitochondrial Ca^2+^ uptake from an UCP3-dependent to a Letm1-dependent mode upon SERCA inhibition in HeLa cells, we reported herein.

Letm1 as well as UCP2/3 were described to accomplish the transfer of Ca^2+^ into mitochondria, while their functioning and contribution to mitochondrial Ca^2+^ uptake is debated (6, 11, 21, 43, 44). Despite strong functional data, the concerns against the idea that Letm1 and UCP2/3 indeed accomplish a transfer of Ca^2+^ across the IMM, are primarily based on the postulation of a unique, ubiquitous Ca^2+^ uniporter being a low sensitive Ca^2+^ channel protein that is activated exclusively by high Ca^2+^ signals. A recently identified protein of the IMM, referred to as MCU, was shown to fulfill some of the criteria that have been expected for a protein that accomplishes mitochondrial Ca^2+^ uniport (12, 13). In agreement with these landmark publications, siRNA-mediated knock-down of MCU attenuated mitochondrial Ca^2+^ signals independently from the mode of Ca^2+^ mobilization in the present study. These observations indicate that MCU is activated over a large range of Ca^2+^ concentration and, hence, contributes to mitochondrial uptake of high and low cellular Ca^2+^ signals, which is, however, in disagreement with the low Ca^2+^ sensitivity of the mitochondrial Ca^2+^ uniport phenomenon (45, 46).

Notably, in several reports elimination of the mitochondrial Ca^2+^ buffering increases cytosolic Ca^2+^ peak and accelerates its decline (13, 47, 48). However, our findings are in agreement with other reports using the same cell type (HeLa) (17, 49, 50) where inhibition of mitochondrial Ca^2+^ uptake failed or only slightly affected cytosolic Ca^2+^ elevation upon stimulation, while the kinetics of decline remained unchanged. Accordingly, these data may indicate that in the cell type used herein, mitochondria have a rather low Ca^2+^ buffer capacity and do not accumulate large proportions of released Ca^2+^, thus, resulting in the lack of strong changes in cytosolic Ca^2+^ signaling by MCU knock-down.

The contribution of the MCU, to mitochondrial Ca^2+^ uptake was shown to be tightly regulated by an associated protein, referred to as mitochondrial calcium uptake 1 (MICU1) (51) that, in contrast to MCU, has Ca^2+^ binding domains. Initially, it was reported that MICU1 facilitates MCU-dependent mitochondrial Ca^2+^ uptake in HeLa (51) and clonal pancreatic beta-cells (25) but not endothelial cells (16). However, in a very recent study a contrary function of MICU1 in HeLa and endothelial cells acting as a gatekeeper and, thus, preventing MCU-dependent mitochondrial Ca^2+^ loads was unveiled (52), thus, indicating that further studies are necessary to understand the definite role of MICU1 in the control of mitochondrial Ca^2+^ uptake. Nevertheless, the intricate regulation of the MCU activity by MICU1 and other associated proteins such as the recently identified mitochondrial calcium uniporter regulator 1 (MCUR1) (53) might explain why the MCU catalyzes mitochondrial Ca^2+^ uptake of both high and low Ca^2+^ signals. From this point of view our data might also indicate that depending on the SERCA activity either UCP2/3 or Letm1 contribute to mitochondrial Ca^2+^ uptake by modulating the activity of MICU1, MCUR1, and/or the MCU. This assumption is further supported by our observation that a double knock-down of either MCU and UCP2/3 or MCU and Letm1 did not further impact mitochondrial Ca^2+^ accumulation. The lack of any further reduction of mitochondrial Ca^2+^ uptake in MCU depleted cells by an additional knock-down of either UCP2/3 or Letm1 also suggest that these proteins might function as upstream regulators of the MCU. However, whether or not the remaining uptake under such conditions indicates a so far unknown additional mitochondrial Ca^2+^ carrier or is due to incomplete diminution of the proteins by the siRNA remains unclear.

The findings that SERCA inhibition abrogates the contribution of UCP3 to mitochondrial Ca^2+^ uptake in HeLa cells was recently interpreted as an indication that UCP3 do not accomplish mitochondrial Ca^2+^ uniport or directly modulate a mitochondrial Ca^2+^ channel (22). The authors suggested that UCP3 reduces SERCA activity by limiting mitochondrial ATP generation, which increases the amount of Ca^2+^ at sites of mitochondrial Ca^2+^ uptake. However such interpretation is in contradiction to the reported lack of uncoupling activity of UCP2/3 (54), findings that overexpression of UCP2/3 boosts mitochondrial ATP generation upon Ca^2+^ mobilization and UCP2/3 contributed to mitochondrial Ca^2+^ uptake also under conditions in which mitochondrial ATP production was prevented (17).This is further supported by the observation that silencing of UCP2/3 failed to hyperpolarize mitochondrial membrane potential (supplemental Fig. S1) However, the present findings confirm the assumption of Demaurex's group and demonstrate that SERCA activity affects mitochondrial Ca^2+^ uptake.

Overall the present study demonstrates that the inhibition of SERCA affects the kinetics of IP_3_-triggered intracellular Ca^2+^ release, and, subsequently, shifts the mode of mitochondrial Ca^2+^ uptake from an UCP3- and MCU-dependent and Letm1-independent toward a Letm1- and MCU-dependent but UCP3-independent route (Fig. 6). These observations indicate that SERCA activity is a crucial determinant for the mode of mitochondrial Ca^2+^ uptake and appoints which proteins of the IMM actually contribute to the transfer of Ca^2+^ into mitochondria.

## Supplementary Material

Supplemental Data

## Footnotes

5 The abbreviations used are:

IMM: inner mitochondrial membrane
[Ca^2+^]_cyto_: cytosolic free Ca^2+^ concentration
Letm1: leucine zipper EF hand-containing transmembrane protein 1
MCUR1: mitochondrial calcium uniporter regulator 1
MICU1: mitochondrial Ca^2+^ uptake 1
[Ca^2+^]_mito_: mitochondrial Ca^2+^ concentration
SERCA: sarco/endoplasmic reticulum Ca^2+^-ATPase
SOCE: store-operated Ca^2+^ entry
UCP2/3: uncoupling protein 2 and 3.
