# Supplementary material for: Molecularly Distinct Routes of Mitochondrial Ca2+ Uptake Are Activated Depending on the Activity of the Sarco/Endoplasmic Reticulum Ca2+ ATPase (SERCA)
Source: J Biol Chem. 2013 Apr 16;288(21):15367–79. doi: 10.1074/jbc.M113.462259 (PMC3663555; doi:10.1074/jbc.M113.462259)
Supplement: Supplemental Data [file supp_288_21_15367__index.html]

Molecularly distinct routes of mitochondrial Ca2+ uptake are activated depending on the activity of the sarco/endoplasmic reticulum Ca2+ ATPase (SERCA) — Molecularly Distinct Routes of Mitochondrial Ca2+ Uptake Are Activated Depending on the Activity of the Sarco/Endoplasmic Reticulum Ca2+ ATPase (SERCA) — SERCA Affects Mitochondrial Ca2+ Uptake — Supplemental Data 

# Molecularly Distinct Routes of Mitochondrial Ca2+ Uptake Are Activated Depending on the Activity of the Sarco/Endoplasmic Reticulum Ca2+ ATPase (SERCA)

## Supplemental Data

**Files in this Data Supplement:**

- Supplemental File (.pdf, 441 KB) - This file has been included in this version to address the referees' comments and to show all data concerning the points raised.
