# Supplementary material for: Molecularly Distinct Routes of Mitochondrial Ca2+ Uptake Are Activated Depending on the Activity of the Sarco/Endoplasmic Reticulum Ca2+ ATPase (SERCA)
Source: J Biol Chem. 2013 Apr 16;288(21):15367–79. doi: 10.1074/jbc.M113.462259 (PMC3663555; doi:10.1074/jbc.M113.462259)

Supplemental Material to

Molecularly distinct routes of mitochondrial Ca^{2+} uptake are activated depending on the activity of the sarco/endoplasmic reticulum Ca^{2+} ATPase (SERCA)

Markus Waldeck-Weiermair[1], András T. Deak[1], Lukas N. Groschner, Muhammad Rizwan Alam, Claire Jean-Quartier, Roland Malli and Wolfgang F. Graier[*]

Institute of Molecular Biology and Biochemistry, Center of Molecular Medicine, Medical University of Graz, 8010-Graz, Austria

Supplemental Figure S1: Mitochondrial membrane potential remains unaffected by silencing of MCU, UCP2/3 or Letm1

HeLa cells were co-transfected with the respective siRNAs and a plasmid encoding for nuclear localization signal-green fluorescent protein in order to allow identification of transfected cells in subsequent experiments. 48 h after transfection cells were loaded with 200 nM tetramethylrhodamine methyl ester (TMRM) for 30 min at room temperature. Experiments were carried out in quench mode as described recently (1). TMRM fluorescence of single cells was recorded and normalized to peak fluorescence intensities during depolarization of $\Delta\Psi_{mito}$ with 2 µM of the protonophore carbonyl cyanide-4-(trifluoromethoxy)phenylhydrazone (FCCP).

Mitochondrial inner membrane potential was determined by measuring TMRM fluorescence after application of siRNAs targeting MCU (n=77), UCP2/3 (n=64), Letm1 (n=79) or a combination of siRNAs targeting both MCU and UCP2/3 (n=72) or MCU and Letm1 (n=79). Cells transfected with scrambled siRNA were used as a control (n=80). Silencing of MCU, UCP2/3 or Letm1 did not alter mitochondrial membrane potential and neither did the combined knock-down of MCU and UCP2/3 or that of MCU and Letm1.

Supplemental Figure S2: Mitochondrial Ca^{2+} extrusion is not affected by the siRNA mediated knockdown of MCU, Letm1, UCP2/3 or MCU in a combination with Letm1 or UCP2/3

HeLa cells were co-transfected with the mitochondrial Ca^{2+} sensor 4mtD1GO-Cam and siRNAs against MCU (siMCU), Letm1 (siLetm1), UCP2/3 (siUCP2/3) or a combination of MCU together with either UCP2/3 (siUCP2/3 + siMCU) or Letm1 (siLetm1 + siMCU). Mitochondrial Ca^{2+} was measured under nominal Ca^{2+} free condition upon stimulation with 100 µM histamine in the absence (***A-E, K***) or presence of 1µM thapsigargin (***F-J, L***). Mitochondrial Ca^{2+} efflux capacity was determined by the slope of the decrease of normalized 4mtD1GO-Cam signals. The drop kinetic of normalized ratio signals starting either at 1.03 (histamine stimulation alone) or at 1.04 (in the presence of thapsigargin) under each individual condition was analyzed over a time period of 20 seconds using linear regression. Cells transfected with a scrambled siRNA were used as Control (Control, grey curves and black regression lines) under both conditions, in the absence (n=18, ***A-E***) or presence (n=15, ***F-J***) of thapsigargin. (***A***) siUCP2/3 (green curve and regression line, n=8); (***B***) siMCU (orange curve and regression line, n=13), (***C***) siLetm1 (brown curve and regression line, n=11), (***D***) siLetm1 + siMCU (violet curve and regression line, n=11), (***E***) siUCP2/3 + siMCU (dark red curve and regression line, n=7), (***F***) siUCP2/3 (n=13), ***(G)*** siMCU (n=15), (***H***) siLetm1 (n=11), ***(I)*** siLetm1 + siMCU (n=7), (***J***) siMCU + siUCP2/3 (n=6). (***K, L***) Respective initial normalized 4mtD1GO-Cam ratio values (1.03 in the absence of thapsigargin and 1.04 in the presence of thapsigargin) were defined as 100 % and the average slopes of the linear regressions were calculated and plotted as percentage per second (%/s).

Supplemental Figure S3: Double knock-down of either MCU and UCP2/3 or MCU together with Letm1 did not further reduce $[Ca^{2+}]_{mito}$

HeLa cells were transfected with a scrambled siRNA (Control, black curves) or siRNA against MCU (siMCU, orange curves) alone or in combination with either siRNA against Letm1 (siLetm1 + siMCU, violet curves) or UCP2/3 (siUCP2/3 + siMCU, dark red curves) together with a plasmid encoding for 4mtD1GO-Cam. (***A***) Cells were stimulated with 100 µM histamine in a nominal Ca^{2+} free solution and simultaneously recorded for mitochondrial *(left panel)* or cytosolic Ca^{2+} *(right panel)*. Average curves of Control (n=21) were compared with that of siMCU (n=17), siLetm1 + siMCU (n=18) or siUCP2/3 + siMCU (n=27). *, $p < 0.05$ vs. Control $[Ca^{2+}]_{mito}$. (***B***) Cells were stimulated with histamine in the presence of thapsigargin and mitochondrial *(left panel)* and cytosolic Ca^{2+} *(right panel)* were simultaneously measured. Statistical analysis was performed for siMCU (n=20), siLetm + siMCU (n=14) and siUCP2/3 + siMCU (n=14) versus Control (n=19). *, $p < 0.05$ vs. Control $[Ca^{2+}]_{mito}$.

References:

1 Deak AT, Groschner LN, Alam MR, Seles E, Bondarenko AI, Graier WF, Malli R. (2012) The endocannabinoid N-arachidonoyl glycine (NAGly) inhibits store-operated Ca^{2+} entry by abrogating STIM1/Orai1 interaction. Journal of Cell Science 2013 (in press) doi: 10.1242/jcs.118075

Supplemental Figure S1

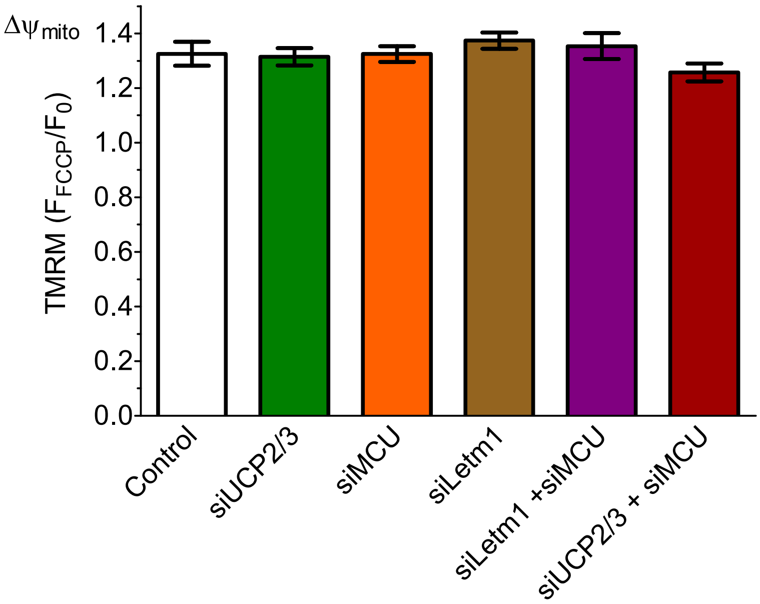

Supplemental Figure S2

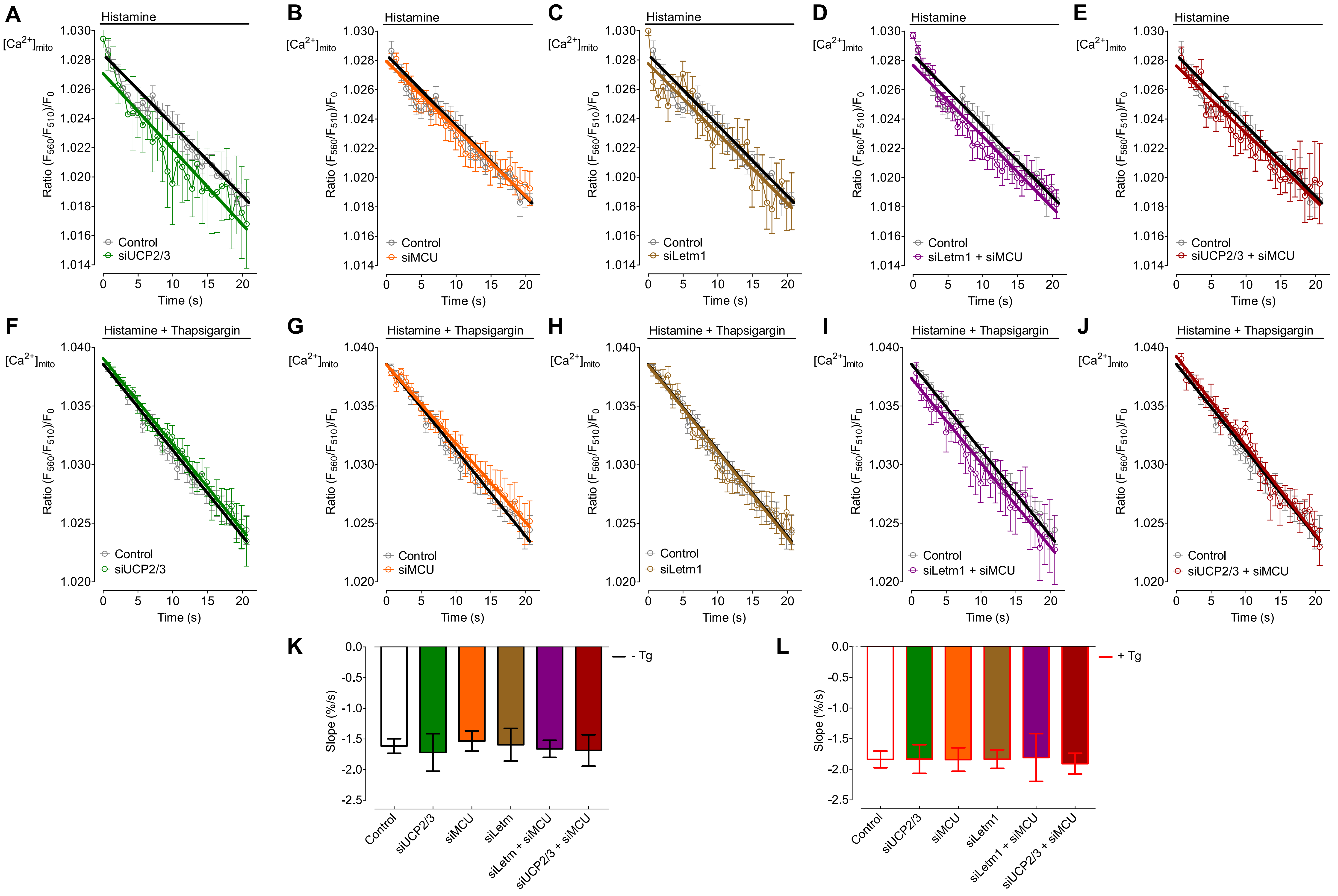

Supplemental Figure S3

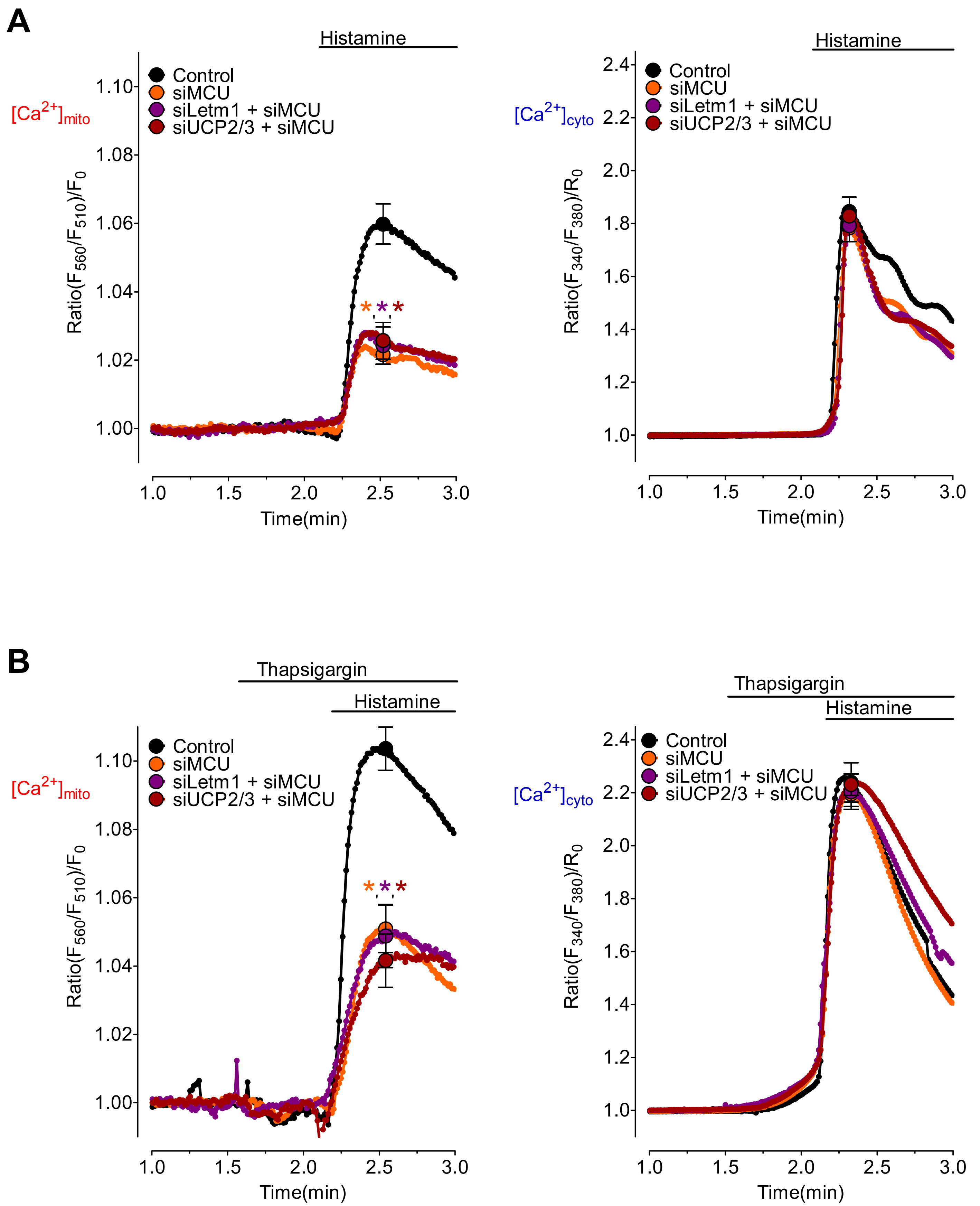

Supplement: Supplemental Data [file supp_M113.462259_jbc.M113.462259-1.pdf]
